# Prevalence and experiences of exclusive breastfeeding among working mothers at a tertiary hospital in Eastern Uganda

**DOI:** 10.1038/s41598-025-30619-9

**Published:** 2025-12-18

**Authors:** Nelson Cherop, Ivan Lyagoba, Proscovia Nabachenje, Annette Jane Namugaya Mugabe, Immaculate Mbwali

**Affiliations:** 1 Department of Nursing, Faculty of Health Sciences, Busitema University, P.O. Box 1460, Mbale City, Uganda; 2 Department of Pediatrics, Faculty of Health Sciences, Busitema University, P.O. Box 1460, Mbale City, Uganda

**Keywords:** EBF, Working mothers, Prevalence, Challenges, Facilitators, Mbale regional referral hospital (MRRH), Health care, Medical research

## Abstract

Exclusive breastfeeding (EBF) is vital for infant health, yet working mothers often struggle to maintain it. In Uganda, particularly at Mbale Regional Referral Hospital (MRRH), evidence on EBF prevalence and the experiences of employed mothers remains limited. This study examined the prevalence of EBF and explored the experiences of working mothers seeking care at MRRH. A cross-sectional mixed-methods design was used. Quantitatively, 221 working mothers with infants aged six months and below completed structured questionnaires. Qualitatively, 10 purposively selected mothers participated in in-depth interviews to provide detailed insights into their EBF experiences. Descriptive statistics summarized quantitative data, while thematic analysis identified patterns within qualitative narratives. EBF prevalence was 68.3%. Four themes emerged: Diverse breastfeeding practices, variable knowledge of EBF, and supportive factors and challenges/barriers to EBF. Mothers described adapting feeding schedules and responding to infant cues to sustain EBF. Knowledge varied, with some emphasizing nutritional and bonding benefits. Support from family members and proximity to the infant facilitated EBF, while work demands, short maternity leave, and inadequate breastfeeding-friendly facilities were major challenges. EBF among working mothers remains sub-optimal. Strengthened workplace policies and broader support systems are essential to enhance EBF continuity among employed women.

## Introduction

EBF is recommended for mothers from the time of delivery until their infants reach six months of age^1^. Globally, approximately 35% of newborns are exclusively breastfed during their first four months of life, with only 38% of infants under six months in developing countries receiving EBF^2^. A previous study identified industrialization and urbanization, particularly work-related challenges as significant barriers to EBF, resulting in a stagnant prevalence of 17% over a decade^2^. However, more recent data indicate an encouraging increase in global EBF rates to 48% among infants under six months^3^.

In Africa, EBF rates vary by region: 57% in Southern Africa, 24% in Central Africa, 46.37% in West Africa, and just 17.6% in East Africa^4^. In Uganda, while 90% of children are breastfed at some point, only 63% of infants under six months are exclusively breastfed^5^. Alarmingly, 29% of children under five years old who are not exclusively breastfed experience stunting^6^. The prevalence of EBF in Uganda decreases with age: 83% among infants aged 0–1 month, 70% at 2–3 months, and 43% at 4–5 months^5^. A mixed-methods study in Eastern Uganda found an EBF prevalence of 63% in the Manafwa district^7^.

Despite a generally high intention of many women to exclusively breastfeed, many face substantial barriers that reduce EBF adherence^8^. These challenges include unsupportive workplace environments, early return to work, psychological stress, and the absence of policies that protect and promote breastfeeding in the workplace^8–10^. Additional obstacles include inadequate family support, conflicting work schedules, household responsibilities, and low breast milk production^1,11^. Working mothers often face difficulties in balancing their dual responsibilities of breastfeeding and employment^12–14^. For mothers working in the formal sector, lack of social protections such as access to maternity leave, job security, and workplace support for breastfeeding remain major obstacles, while those in informal employment frequently experience even greater challenges, including the absence of legal protections, and lack of structured workplace support^15^. Furthermore, inadequate community and media support, as well as partner opposition to breastfeeding, exacerbate these challenges^16^.

Although some literature exists on EBF in Uganda, there is limited information specific to working mothers in urban hospital settings such as Mbale Regional Referral Hospital (MRRH). This study aimed to assess the prevalence of exclusive breastfeeding and to explore the experiences of working mothers attending MRRH. The findings aim to inform targeted interventions and policies to promote EBF among employed mothers and improve infant health outcomes. In Uganda, most women are engaged in both formal and informal employment, yet workplace environments often lack breastfeeding-friendly policies or infrastructure. For instance, many institutions provide only the statutory 60 working days of maternity leave, after which mothers are compelled to return to work, often without designated lactation spaces. In Eastern Uganda, research from Mbale district indicates that mothers frequently discontinue EBF prematurely due to conflicting work demands and the absence of institutional support^17^. This situation places infants at increased risk of malnutrition, stunting, and frequent illness. This highlights the urgent need to explore the EBF experiences of mothers in the region.

## Methods and materials

### Study aim

This study aimed to assess the prevalence of exclusive breastfeeding and to explore the experiences of EBF among working mothers seeking healthcare services at MRRH.

### Study design and study setting

A cross-sectional study design employing a mixed-methods approach was used to collect both quantitative and qualitative data. The study was conducted at MRRH, a public tertiary-level facility located in Mbale City, northeast of Kampala, Uganda. The hospital has a bed capacity of approximately 500 and serves over 16 districts and one city. It receives about 300 breastfeeding mothers, of which around 200 are working mothers seeking health care services at Young Child Clinic (YCC) in a month. The hospital provides maternal and child health services, including immunization, nutrition counseling, and health education on EBF. The study was carried out in the YCC and the Pediatric Ward, where services are provided by qualified healthcare professionals including nurses, midwives, doctors, and pediatricians. Notably, MRRH launched a breast milk bank on May 17, 2024, to support EBF efforts.

### Study population, sample size and data collection procedure

The study population comprised 221 breastfeeding working mothers (quantitative sample) determined using the Kish and Leslie (1965) and 10 participants (qualitative sample) seeking healthcare services at YCC and Pediatrics unit at MRRH. Eligible participants were working mothers with infants aged six months or younger. Mothers with babies having cleft palate and lip were excluded from the study since the condition interrupts with breastfeeding.

Quantitative data was collected through simple random sampling. Upon arrival at the study sites, the investigators identified and randomly selected eligible mothers. After obtaining informed consent, structured questionnaires were administered by the investigator.

For the qualitative component, purposive sampling was used to select participants. In-depth interviews were conducted with working mothers who provided informed consent. Open-ended questions were asked based on a pre-developed interview guide aligned with the research objectives. Each interview lasted approximately 15 min. Ethical approval was obtained from the Busitema University, Faculty of Health Sciences Ethics and Research Committee under approval number BUFHS-2024-215.

### Data analysis

Quantitative data were cleaned in Microsoft Excel and analyzed using SPSS version 15.0. Descriptive statistics, including means, frequencies, and percentages, summarized participant characteristics. The prevalence of EBF was determined by dividing the number of exclusively breastfeeding working mothers by the total sample and multiplying by 100%. Qualitative data from interviews were transcribed and analyzed thematically using Atlas software. Codes were identified, grouped into subthemes, and categorized into broader themes representing the challenges and enablers of EBF. Data triangulation was used to enhance the validity of findings from both quantitative and qualitative sources.

## Results

### Quantitative results

#### Description of study participants

**Table 1:** Socio-demographic characteristics of the participants.

	Currently breastfeeding exclusively?		
	No	Yes	Total
	*n* = 70	*n* = 151	*n* = 221
Age (years) (mean = 27.2 years)			
Below 20	8 (11.4%)	24 (15.9%)	32 (14.5%)
20–34	51 (72.9%)	104 (68.9%)	155 (70.1)
Above 34	11 (15.7%)	23 (15.2%)	34 (15.4%)
Address			
1. Rural	21 (30.0%)	41 (27.2%)	62 (28.1%)
2. Urban	49 (70.0%)	110 (72.8%)	159 (71.9%)
Level of education			
1. No formal education	5 (7.1%)	4 (2.6%)	9 (4.1%)
2. Primary education	12 (17.1%)	32 (21.2%)	44 (19.9%)
3. Secondary/tertiary education	53 (75.7%)	114 (76.2%)	168 (76.0%)
Marital status			
Married	57 (81.4%)	116 (76.8%)	173 (78.3%)
Not married (single, divorced, widowed):	13 (18.6%)	35 (23.1%)	48 (21.8%)
Nature of employment			
Formal employment	25 (35.7%)	42 (27.8%)	67 (30.3%)
Informal employment	45 (64.3%)	109 (72.2%)	154 (69.7%)
Infant’s health status			
Excellent	2 (2.9%)	21 (13.9%)	23 (10.4%)
Good	67 (95.7%)	130 (86.1)	197 (89.2)
Poor	1 (1.4%)	0 (0.0%)	1 (0.5%)
Mother’s confidence in breastfeeding			
Very confident	2 (2.9%)	28 (18.5%)	30 (13.6%)
Confident	34 (48.6%)	69 (45.7%)	103 (46.6%)
Not confident	34 (48.6%)	54 (35.8%)	88 (39.9)
Mode of delivery			
Cesarean section	11 (15.7%)	38 (25.2%)	49 (22.2%)
Spontaneous vaginal delivery.	59 (84.3%)	113 (74.8%)	172 (77.8%)
Place of delivery			
Health center	63 (90.0%)	147 (97.4%)	210 (95.0%)
Home	7 (10.0%)	4 (2.6%)	11 (5.0%)
Who delivered the baby?			
Healthcare provider	62 (88.6%)	147 (97.4%)	209 (94.6%)
Traditional birth attendant	8 (11.4%)	4 (2.6%)	12 (5.4%)

#### Prevalence of EBF among working mothers

**Fig. 1:**
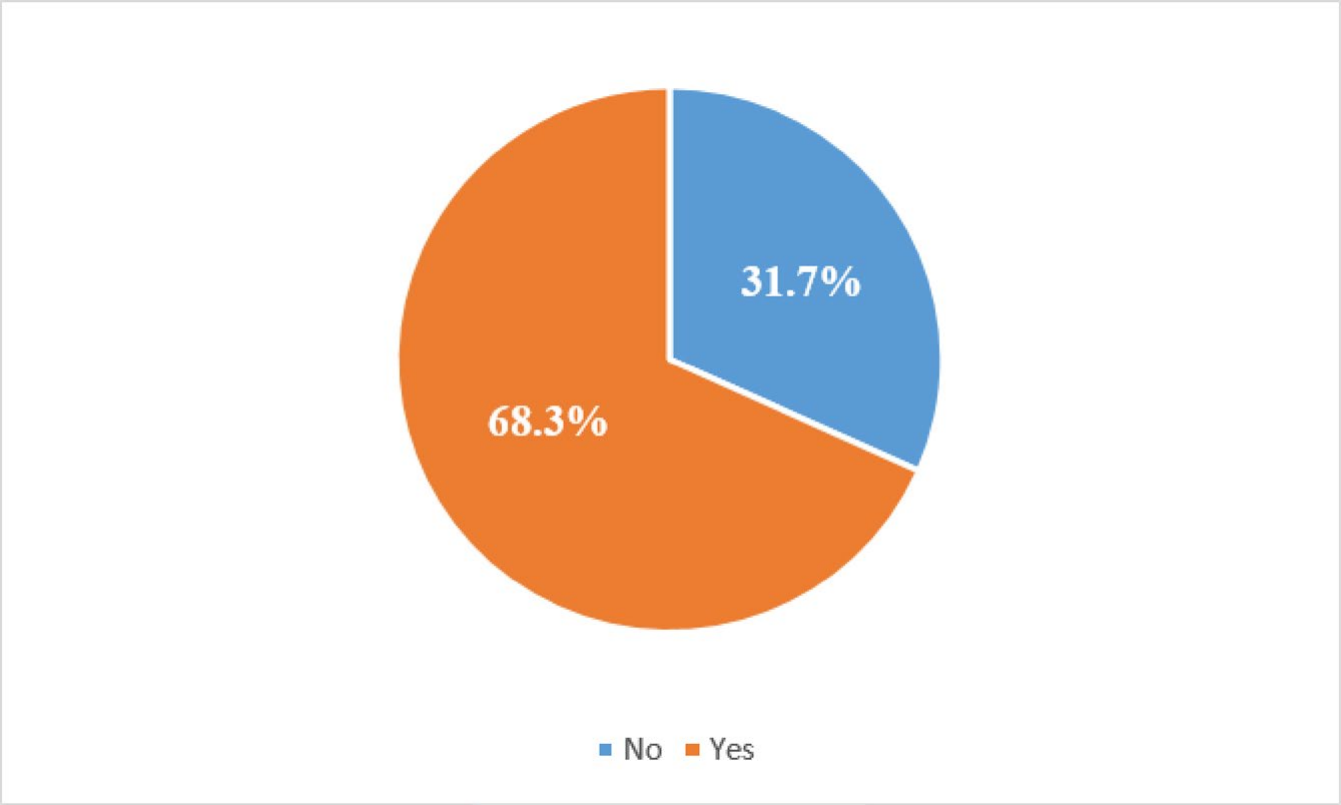
Prevalence of EBF breastfeeding among working mothers.

The prevalence of EBF is 68.3% obtained from (151/221) *100 as indicated in Fig. 1 below.

Among the 151 mothers who practiced exclusive breastfeeding, the majority were Bamasaba (41.1%), followed by Itesot (17.2%) and Sabiny (8.6%), with smaller proportions among Baganda (6.6%), Acholi (2.0%), and other tribes (24.5%). Most women (72.2%) practicing EBF were in informal employment. Anglicans were the majority (59.6%), followed by Catholics (33.8%), with fewer being Muslims (6.0%) and Born Again (0.7%). These findings indicate that while EBF was practiced across different ethnic and religious groups, it was more common among the Bamasaba and Anglicans, reflecting the demographic dominance of these groups in the study setting (Table 1).

#### Qualitative results

**Table 2:** Themes, subthemes, codes, and meaning units representing the participant submissions on EBF.

Themes	Subthemes	Codes	Meaning units
1.0: Diverse breastfeeding practices	1.1 Frequency of breast feeding	Adjusting according to daily work schedules.	“…*then I express some milk*,* then leave it in the bottle for my housemaid and when I come back from work*,* then I start from where*,* I stopped*,* I continue breastfeeding him…”*
		Scheduled breast feeding before/after work	*“…breast feed him when I am about to go to work…in the evening when I come back and then the next day before I go work”*
	1.2 Responsiveness and proactiveness in breastfeeding	Baby waking up to breast feed.	*“…even when she is sleeping*,* I can wake her up to breastfeed”*
		On-demand night breast feeding	*“At night…when the baby cries*,* I can wake up and give him breastfeed*
		Responsive to breastfeeding cues.	*“…when the baby starts crying the helper brings to me for breastfeeding…”*
			“…*some signs that I see on my baby that will show me my baby wants to breastfeed like putting the thumb into the mouth.”*
			*“Sometimes the baby starts making noise*,* and I don’t wait for her to cry”.*
2.0 Variable knowledge of EBF	2.1 Knowledge driven EBF	Giving only breast milk	“*EBF is when you feed the baby only on breast milk…don’t give any food.”*
		Duration of EBF	*“EBF…takes six months*,* without adding other food”*
	2.2 Living the benefits of breast milk	Rich in vital nutrients for baby growth.	*“As a breastfeeding mother*,* I need to know more importance of breast milk*,* because what I know is that it has some nutrients that facilitate the growth of our babies.”*
		Strengthens immunity	*“…they also get immune from our body and they don’t fall sick frequently in that early age.”*
		Bonding of mother to baby	*“…this…bonding. When the mother is with the baby…creates a very good bonding. And can recognize me as the mother.”*
3.0: Supportive factors for EBF	3.1 Family support	Spouse assistance	*“My husband can support me at home*,* taking care of other things while I breastfeed the baby.”*
		Assistance from other people in the house	*“…When I have people around me to support in doing other home activities*,* I can now only pay attention to my child and exclusively breastfeed…”*
		Help from the Maid	*“I have a maid who helps with other work while I focus on breastfeeding.”*
	3.2 Proximity to workplace	Work-place near home.	*“…my mobile money outlet is near where I stay…sometimes I will be running home…I check on him…”*
		Flexible working days	*“They have given us days for market*,* like Monday…then Wednesday*,* then Friday…so the remaining days*,* at least I breastfeed this child to the maximum.”*
4.0 challenges and barriers to EBF.	4.1 Time-consuming and exhausting demands from work	Strictly engaging work.	*“Sometimes it is hard because I can be busy. The busy nature of my work makes it difficult for me to breastfeed exclusively.”*
		Interruptions from work-related stress	*“Then after coming back from work and you are very tired*,* the tiredness takes me over…I don’t always get that time to breastfeed the baby.”*
	4.2 Lack of workplace breastfeeding support facilities and policies	No designated breastfeeding space at workplace.	*“…we don’t have rooms which can help to facilitate*,* make us keep our children*,* so that we can breastfeed them as required.”*
		Employer restrictions on babies.	*“…our bosses*,* they don’t allow us to bring children to the workplace and I just leave my child to stay with the baby sitter.”*

### Theme 1: breastfeeding practices

Breastfeeding practices included mothers’ adjustments to breastfeeding frequency and attentiveness to infant cues, despite challenges.

#### Subtheme 1.1: frequency of breastfeeding

Mothers adopted flexible breastfeeding schedules to accommodate their work routines, adjusting feeding practices to balance professional and caregiving responsibilities.

*“…then I express some milk…leave it in the bottle for my housemaid and when I come back from work*,* then I start from where I stopped*,* I continue breastfeeding him.”* (30-year-old Para 5, mother 12).

*“At night… I really compensate…I really give him to breastfeed so that he can be able to gain up.”* (25-year-old Prime Para, mother 6).

#### Subtheme 1.2: responsiveness and proactiveness in breastfeeding

Mothers described being highly responsive to their babies’ feeding needs, often initiating breastfeeding at the first sign of restlessness (Table 2). Even during the night or when the baby was asleep, they remained alert and committed to waking up or waking the baby to ensure consistent breastfeeding.

*“Sometimes the baby starts making noise*,* and I don’t wait for her to cry…When I breastfeed her*,* like after 30 minutes*,* an hour*,* even when she is sleeping*,* I can wake her up to breastfeed.”* (25-year-old Para 2, Mother 3).

*“At night…when the baby cries*,* I can wake up and give him breastfeed.”* (29-year-old Para 3, Mother 11).

Mothers also exhibited attentiveness to their babies’ breastfeeding cues, often feeding being the first sign of hunger or restlessness rather than waiting for crying, which they interpreted as an advanced signal of need. Many mothers described being attuned to signs like thumb-sucking or soft noises, indicating a proactive approach to breastfeeding. For instance, one participant shared how observing her baby’s thumb-sucking as a cue to start feeding helped to avoid escalation to crying. This early response approach reflects mothers’ understanding of their infants’ unique hunger cues and their determination to meet these needs promptly.

*“I breastfeed my baby during the day because I get to know about this when my baby cries and some signs that I see on my baby that will show me my baby wants to breastfeed like putting the thumb into the mouth.”* (25- year-old Prime Para. Mother 6).

*“Sometimes the baby starts making noise*,* and I don’t wait for her to cry…”* (25-year-old Para 2, Mother 3).

### Theme 2: variable knowledge of EBF

The participants demonstrated varying levels of knowledge regarding EBF, which informed their feeding practices. Mothers express an understanding of EBF’s requirements and benefits, recognizing the exclusive provision of breast milk for the first six months of life.

#### Subtheme 2.1: Knowledge-driven EBF

Participants generally defined EBF as feeding their babies only with breast milk for a period of six months, excluding any supplementary foods or drinks. Mothers showed a clear understanding of EBF, what it means and how for long it should be carried. For these mothers, EBF represented a commitment to their infants’ health, with breast milk viewed as the sole source of nutrition during this formative period. This understanding is echoed in one participant’s straightforward explanation that EBF means “only on breast milk,” reflecting her belief in the importance of this practice for her infant’s development.

*“EBF is when you feed the baby only on breast milk…don’t give any food.”* (29-year-old Para 3, mother 11).

*“EBF…takes six months*,* without adding other food.”* (27-year-old Para 3, Mother 5).

#### Subtheme 2.2: living the benefits of breast milk

Some mothers shared understanding of the health benefits of breast milk. Their beliefs reflected a connection between exclusive breastfeeding and the baby’s improved growth, development, and early immunity and bonding with the mother.

*“Because what I know*,* breast milk has a lot of nutrients which support the baby’s growth and development…they also get immune from our body and they don’t fall sick frequently in that early age.”* (29-year-old Para 3, mother 11).

This experience is further evidenced by all mothers who were exclusively breastfeeding reporting that their children were in good or excellent health status.

### Theme 3: supportive factors for EBF

Mothers benefited from various supports that helped facilitate EBF. These supports ranged from family assistance with daily tasks to workplace flexibility that allowed them to feed their infants as needed.

#### Subtheme 3.1: family support

Participants emphasized that family support, particularly from spouses and house helpers, is crucial for enabling EBF. Assistance with household tasks provided mothers the time and space needed for uninterrupted breastfeeding, highlighting the shared responsibility in promoting the infant’s wellbeing.

*“My husband can support me at home*,* taking care of other things while I breastfeed the baby.”* (25-year-old Para 2, Mother 3).

*“…When I have people around me to support in doing other home activities*,* I can now only pay attention to my child and exclusively breastfeed…”* (24-year-old Prime Para, Mother 4).

#### Subtheme 3.2: proximity to workplace

Some women in the informal employment sector reported the proximity of their homes to their workplaces that it allowed them to quickly check on their babies even when busy running work errands. This proximity helped them balance work and breastfeeding effectively.

*“…my mobile money outlet is near where I stay…sometimes I will be running home…I check on him…”* (24-year-old Prime Para, Mother 4).

### Theme 4: Challenges and barriers to EBF

#### Subtheme 4.1: Time-consuming and exhausting demands from work

Women revealed how structural demands of employment, such as long hours, physically exhausting work, and lack of designated breaks, directly undermined their ability to practice EBF.

*“Sometimes it is hard because I can be busy. The busy nature of my work makes it difficult for me to breastfeed exclusively.”* (24-year-old Prime Para, mother 1).

*“Then after coming back from work and you are very tired*,* the tiredness takes me over…I don’t always get that time to breastfeed the baby.”* (30-year-old Para 5, Mother 12).

#### Subtheme 4.2: lack of workplace breastfeeding support facilities and policies

Several limitations hindered mothers’ ability to sustain EBF while fulfilling work responsibilities. The lack of designated breastfeeding facilities and prohibition to bring children to workplaces by some employers made it more difficult for mothers to practice EBF.

*“…we don’t have rooms which can help to facilitate*,* make us keep our children*,* so that we can breastfeed them as required.”* (29-year-old Para 3, mother 11).

*“…our bosses*,* they don’t allow us to bring children to the workplace and I just leave my child to stay with the babysitter.”* (Mother 11).

## Discussion of results

The study findings on the prevalence of EBF among working mothers at MRRH align with existing research showing moderate EBF rates. A significant portion of mothers (68.3%) exclusively breastfed, with higher prevalence among women aged 26–34 years, consistent with findings in Kampala, Uganda, where EBF was observed around 43% among younger mothers^18^. This suggests age-related differences in EBF practices, potentially due to greater awareness and confidence in breastfeeding among middle-aged mothers.

The findings on breastfeeding practices revealed how working mothers adapt flexible breastfeeding schedules and responsiveness to infant cues as strategies to sustain exclusive breastfeeding. This adaptation shows the resilience and dedication of mothers who balance employment with childcare. Similar findings have been reported in Ghana and South Africa, where working mothers creatively reorganize feeding to sustain EBF despite demanding schedules^1,8^. These experiences reflect the reality that most workplaces in urban settings in Uganda lack breastfeeding-friendly policies or infrastructure, leaving mothers to improvise by expressing milk or compensating through night feeding. Such challenges, while demonstrating maternal breastfeeding commitment, expose mothers to fatigue and emotional strain, which could affect both maternal well-being and infant care. The absence of structural support in most similar Ugandan workplaces, where informal employment predominates, indicates that without deliberate policy interventions such as flexible working hours and designated lactation spaces, many mothers will continue to encounter difficulties in sustaining EBF^17,19^.

Mothers’ varying levels of knowledge about EBF illustrate how awareness shapes practices and influences infant outcomes. While some demonstrated a clear understanding of the recommended six months of exclusive breastfeeding and the nutritional benefits of breast milk, others probably lacked comprehensive awareness, which might have compromised adherence. Similar variations in maternal knowledge have been reported in Uganda and Ghana, where knowledge gaps remain a key predictor of early discontinuation of EBF^4,5,12^. These findings show the need for consistent, culturally sensitive health education, especially during antenatal and postnatal visits, since community beliefs and misinformation sometimes discourage sustained EBF. Mothers in this study who recognized breast milk’s role in boosting immunity and fostering bonding were more motivated to sustain the practice, echoing the importance of knowledge-driven confidence as an enabler of breastfeeding^6,7^. Strengthening health worker-led counseling and community sensitization in communities can bridge these knowledge gaps and empower mothers to practice EBF for the recommended duration.

The barriers to EBF identified were work constraints, insufficient milk supply, and lack of support align with findings by^15^ who highlighted that limited workplace accommodations can restrict a mother’s ability to maintain EBF. Work-related constraints remain one of the most challenging obstacles, as many mothers must return to work without the necessary support systems for breastfeeding. The absence of flexible working hours and on-site daycare facilities also highlights gaps in workplace breastfeeding policies, echoing studies in Kenya and Tanzania that reported that supportive breastfeeding policies, including flexible scheduling and provision of lactation rooms have positive impacts on EBF rates^20^.

The findings demonstrated a need for improved social and family support systems. Studies have shown that family and community support, along with reduced societal stigma, are crucial for EBF adherence^8^. This literature emphasizes that EBF success among working mothers often hinges on holistic support that includes workplace accommodations, family involvement, and cultural shifts like.

Mothers in this study also suggested workplace adjustments that could further facilitate EBF. Flexible working hours and onsite daycare facilities were among the most requested changes, which aligns with studies showing that these accommodations significantly reduce logistical barriers and enable mothers to sustain EBF^20,21^. Additionally, extended maternity leave and provision of lactation rooms emerged as potential solutions. Longer maternity leaves have been shown to correlate with higher EBF rates, providing mothers sufficient time to establish breastfeeding routines before returning to work^19^. Lactation rooms, in particular, create a safe, private space for milk expression, which is crucial for maintaining milk supply and EBF while working^15^. These suggested adjustments may inform workplace policies in similar settings to support working mothers and improve EBF rates.

### Limitations

Being a cross-sectional study, this research captured data at a single point in time, which limits the ability to assess changes in EBF practices over time or to establish causal relationships. Additionally, the study was conducted at a single facility, MRRH, which may not fully reflect the experiences of all working mothers in the broader region. Purposive sampling may have introduced some inaccuracy, as key knowledgeable mothers could have been left out, limiting the diversity of perspectives. Since the interviewer was a health worker, social desirability bias may also have influenced responses during interviews, as participants could have provided answers they perceived as acceptable to researchers. Furthermore, the relatively small qualitative sample size could have restricted the depth of variability in perspectives captured. These limitations suggest caution when interpreting the findings, which should be viewed as exploratory and context-specific rather than generalizable to the wider Ugandan population.

## Conclusion

EBF prevalence among working mothers was relatively high at 68.3%, yet the experiences of women in both formal and informal employment revealed persistent variance in knowledge and gaps in support, and workplace accommodations. While many mothers relied on flexible feeding routines and infant-led cues to sustain breastfeeding, variations in understanding of EBF and differing levels of support shaped their practices. Women in informal employment often benefited from greater flexibility and proximity to their infants, whereas those in formal jobs faced stricter schedules, shorter maternity leave, and limited breastfeeding-friendly spaces, all of which constrained their ability to maintain EBF. Overall, the study highlights the need for strengthened workplace policies, extended maternity protection, and enhanced community and family support systems to improve EBF outcomes across formal and informal employment sectors.

## Data Availability

The datasets generated and/or analysed during the current study are available from the corresponding author on reasonable request.

## Abbreviations

EBF: Exclusive breastfeeding
MRRH: Mbale regional referral hospital
YCC: Young child clinic
